# Glu89Gln transthyretin-related amyloidosis in Italy and Bulgaria: does geographic area influence phenotype beyond the shared mutation?

**DOI:** 10.1186/1750-1172-10-S1-P23

**Published:** 2015-11-02

**Authors:** Christian Gagliardi, Mariana Gospodinova, Simone Longhi, Agnese Milandri, Mario Cinelli, Ivailo Tournev, Fabrizio Salvi, Claudio Rapezzi

**Affiliations:** 1 Diagnostic and Specialty Medicine – DIMES, Alma Mater Studiorum, University of Bologna, Cardiology, 40138, Bologna, Italy; 2 University Hospital Alexandrovska, Clinic of Cardiology, 1000, Sofia, Bulgaria; 3 University Hospital Alexandrovska, Clinic of Neurology, 1000, Sofia, Bulgaria; 4 Bellaria Hospital, Neurology, 40100, Bologna, Italy

## Background

Glu89Gln transthyretin (TTR) variant is a well-known cause of systemic amyloidosis with a cardiologic, neurologic or mixed phenotype. Even though Glu89Gln transthyretin (TTR) variant has been described worldwide, it remains unknown whether geographical area influences the phenotypic expression of the disease (as happens with the Val30Met mutation, which is known to manifest with different phenotypes in different geographical contexts). We hypothesized that significant phenotypic differences exist between patients with Glu89Gln-related amyloidosis according to the specific geographic origin.

## Methods

We retrospectively analysed and compared the clinical, electrocardiographic and echocardiographic findings of 64 patients with Glu89Gln TTR-related amyloidosis from Italy and Bulgaria.

## Results

Despite a similar age at diagnosis of the disease (55 [52-60] years) patients in Bulgarian cohort have a mixed phenotype. A more severe left ventricular wall thickening was present in Bulgarian cohort compared to Italian one (17 [16-18] mm vs 15 [13-16], p=0.009) with a normal ejection fraction in all cases.

## Conclusion

This is the largest series so far of patients with Glu89Gln TTR-related amyloidosis systematically analysed. Overall, disease onset is late with a mixed phenotypic expression. Despite a similar age at onset and a predominance of neurological routes, Bulgarian patients showed a more pronounced cardiomyopathy.

